# Vanadium as a Medicine

**Published:** 1900-04

**Authors:** John L. Bradley

**Affiliations:** Mercersburg, Pa.


### VANADIUM AS A MEDICINE.

By JOHN L. BRADLEY, V.S.,
MERCERSBURG, PA.

Vanadium has been known chiefly to chemists, since its discovery early in this century, as a rare elementary substance, although it occurs quite widely, notably in Mexican lead, in some iron ores, in anthracite coal, and in the furnace slag of certain reducing processes, whence it is now generally obtained. Between 1880 and 1886 Messrs. Witz and Osmond investigated the chemical properties of this substance very thoroughly. These are curious enough to merit notice particularly from the veterinary profession. Dr. Laran, of Paris, who describes his researches in an article in *La Science Francaise*, March 2, says: "Witz and Osmond were the first to show that vanadium, or rather its compounds for vanadium, has not yet been obtained in perfect purity, placed in the presence of an oxidizing body and an organic substance capable of oxidation, has the property of serving as a carrier of oxygen from the former to the latter, and that its office ceases only with the complete reduction of the oxidizing substance."

It has been asked whether this property of vanadium may not be used to oxidize the hæmoglobin of the blood. Dr. Laran's investigations along this line have shown, so he tells us, that vanadic acid is the only compound of vanadium that will answer this purpose, and he has succeeded in obtaining it in a chemically pure and standard form, something that had not been accomplished hitherto.

Having established by experiments on animals the fact that the acid in small doses has no injurious effects, but will benefit those diseases that need oxidation of the blood, such as anæmia, tuberculosis, and all maladies dependent on defective nutrition, should, it seems, be relieved by this treatment. Without detailing the various theories of these diseases, it will suffice to note that iron enters into the composition of the hæmoglobin of the blood, and does the duty of taking up the oxygen of the air we breathe and fixing it in the cells of the organism, and that the only cures of tuberculosis that have been effected have been brought about by causing the patients to breathe, in high altitudes, air surcharged with oxygen while giving to them continually an excess of nutrition. "Vanadic acid plays the same rôle as iron, but in a greater degree; it increases the appetite considerably, and by this means makes over-feeding easy and perfectly natural," not only in such cases as anæmia and tuberculosis has the vanadic acid treatment proved a practical success, Dr. Laran writes, and he leaves little doubt that he has discovered a useful addition to the materia medica.

---

DURING the year 1898 there were 1380 head of horses attacked with glanders in Great Britain.

